# *LncRNA BANCR* Promotes Endometrial Stromal Cell Proliferation and Invasion in Endometriosis via the *miR-15a-5p*/TRIM59 Axis

**DOI:** 10.1155/2022/9083822

**Published:** 2022-10-10

**Authors:** Lixue Liu, Ru Bai, Debang Li, Bai Dai, Ya Tuo

**Affiliations:** ^1^Department of Reproductive Medicine, The Affiliated Hospital of Inner Mongolia Medical University, Hohhot, Inner Mongolia 010050, China; ^2^Inner Mongolia Medical University, Hohhot, Inner Mongolia 010050, China

## Abstract

Long non-coding RNA (LncRNA) emerges as a regulator in various diseases, including endometriosis (EM). This study aims to uncover the role of *long non-coding RNA BRAF-activated non-protein coding RNA* (*lncRNA BANCR*)-mediated competing endogenous RNA mechanism in endometrial stromal cell (ESC) proliferation and invasion in EM by regulating *miR-15a-5p*/TRIM59. ESCs were isolated from eutopic and ectopic endometrial tissues, followed by the determination of Cytokeratin 19 and Vimentin expressions in cells. Then, expressions of *lncRNA BANCR*, *microRNA (miR)-15a-5p*, and tripartite motif-containing 59 (TRIM59) in tissues and cells were determined by real-time quantitative polymerase chain reaction or Western blot assay, and cell proliferation and invasion were evaluated by cell counting kit-8 and transwell assays. After that, the subcellular localization of *lncRNA BANCR* and binding of *miR-15a-5p* to *lncRNA BANCR* or TRIM59 were analyzed. *LncRNA BANCR* was upregulated in ectopic endometrial tissues and ectopic ESCs (Ect-ESCs). Silencing *lncRNA BANCR* suppressed Ect-ESC proliferation and invasion. *LncRNA BANCR* inhibited *miR-15a-5p* to promote TRIM59 expression. *miR-15a-5p* downregulation or TRIM59 overexpression both reversed the effects of silencing *lncRNA BANCR* on Ect-ESC proliferation and invasion. In summary, our findings suggested that *lncRNA BANCR* facilitated Ect-ESC proliferation and invasion by inhibiting *miR-15a-5p* and promoting TRIM59.

## 1. Introduction

Endometriosis (EM) is termed a complex gynecological disorder, diagnosed by the presence of endometrial-like tissue, glands, and stroma outside the uterine cavity [1]. EM will affect about 10% of the female population and not only can it result in heavy social and economic burdens, including, pain, infertility, depression and anxiety, health care costs, and indirect productivity loss, but data are accumulating that malignant transformation is an important consideration [2, 3]. EM is a benign disease similar to malignancy in some perspectives, including estrogen-stimulated proliferation, recurrence, and tendency to metastasis [4]. Pathologically, endometrial stromal cells (ESCs), the major resident cells in human endometrium, are switched to invasive and proliferative phenotypes under the stimulation of cytokines and chemokines, thus exacerbating EM progression [5, 6]. Most current therapies focus on alleviation of pain symptoms and prevention of recurrence but lack the curable intent [7]. Therefore, it is prudent to explore novel molecules sensitive to EM proliferation and invasion, in an effort to improve the prognosis of EM patients.

Long non-coding RNA (LncRNA), the well-studied RNA molecules that regulate multiple cellular and biological processes, are documented to participate in hormone response, behaviors of ESCs, autophagy, and immune disorder in the context of EM, thus affecting EM progression [8]. *LncRNA BRAF-activated non-protein coding RNA (BANCR)*, a well-established oncogenic lncRNA in human cancers, is involved in cell proliferation, migration, invasion, apoptosis, and epithelial to mesenchymal transition [9, 10]. More importantly, *lncRNA BANCR* inhibitor is potent to reduce the volume of eutopic endometrium in animal models by suppressing the production of angiogenic factors [11]. However, the expression pattern and role of *lncRNA BANCR* in ectopic ESCs (Ect-ESCs) remain indistinct.

Some lncRNAs can act as competing endogenous RNAs (ceRNAs) to compete for the microRNA (miRNA) binding sites by means of partial complementarity and further modulate endogenous mRNAs [12]. Especially, *lncRNA BANCR* could serve as a sponge for various miRNAs in cancers, such as *miR-590-5P*, *miR-195-5p*, and *miR-338-3p* [13–15]. In this study, our bioinformatics and experimental data suggested a correlation between *lncRNA BANCR* and *miR-15a-5p*/tripartite motif-containing 59 (TRIM59). *miR-15a-5p* is a non-coding RNA that binds to its complementary messenger RNA (mRNA) and inhibits mRNA translation by regulating mRNA degradation [16, 17]. Of note, *miR-15a-5p* has been demonstrated to show downregulated expression patterns in endometriotic tissues and Ect-ESCs-derived extracellular vesicles [18, 19], and inhibition of *miR-15a-5p* promotes ESC migration and invasion [20]. Moreover, TRIM59, a member of the TRIM protein family that is associated with autophagy, immunity, anti-virus, and carcinogenesis and plays roles in the pathogenesis of osteoarthritis, sepsis, and myocardial ischemia-reperfusion injury [21–25], and TRIM59 overexpression can facilitate the invasion of ectopic ESCs (Ect-ESCs) [26]. Nevertheless, the ceRNA network of *lncRNA BANCR*/*miR-590-5p*/TRIM59 in EM has not been studied before and warrants further investigation.

Taking the aforementioned associations into consideration, we raised a hypothesis that *lncRNA BANCR* mediates a ceRNA network with *miR-15a-5p*/TRIM59 to affect Ect-ESC proliferation and invasion. In this manner, the present study sought to investigate the molecular mechanism of *lncRNA BANCR* in EM, hoping to provide a novel theoretical reference for EM treatment.

## 2. Methods and Materials

### 2.1. Acquisition of Clinical Samples

A total number of 20 EM patients (28–40 years old) who received tissue resection in The Affiliated Hospital of Inner Mongolia Medical University were included in this study for the collection of eutopic and ectopic endometrial tissues. The procedures of sample collection were ratified by the medical ethics committee of The Affiliated Hospital of Inner Mongolia Medical University and conformed to the *Declaration of Helsinki*. The written informed content was signed by each patient. The collected samples were stored in liquid nitrogen and kept at –80°C before the subsequent uses.

Inclusion criteria of EM patients were as follows [27]: (1) at reproductive age (19–45 years old); (2) at the proliferative phase of the menstrual cycle; (3) EM was confirmed by laparoscopic surgery and postoperative histological examination. Exclusion criteria of EM patients were as follows: (1) with any history of malignancy; (2) with autoimmune or metabolic disorders; (3) taking any hormonal medications or dietary supplements within the last three months before the surgery; (4) pregnancy or lactation.

### 2.2. Isolation of Primary Human Endometrial Stromal Cells (hESCs)

hESCs were obtained from eutopic and ectopic endometrial tissues of EM patients in The Affiliated Hospital of Inner Mongolia Medical University. The procedures of cell isolation were as follows: firstly, endometrial tissues were shredded, detached with 4% collagenase (60 min), and centrifuged (500 × *g*, 5 min); secondly, the cell suspensions were centrifuged (3000 × *g*, 10 min); thirdly, cell precipitates were resuspended in Dulbecco's modified Eagle medium (DMEM) supplemented with 10% fetal bovine serum (FBS, Gibco, Carlsbad, CA, USA), 100 U/mL penicillin, and 100 mg/L streptomycin (Solarbio, Beijing, China). Then, isolated ESCs were placed in an incubator and kept at 37°C, and the medium was renewed after the first 24 h of cell culture and then replaced every 2–3 days.

### 2.3. Immunocytochemistry

Cells were fixed with 4% paraformaldehyde, washed with phosphate buffer saline (PBS), and incubated overnight with rabbit monoclonal antibody Cytokeratin 19 (CK19, ab52625, 1 : 400, Abcam, Cambridge, MA, USA) or rabbit monoclonal antibody Vimentin (ab92547, 1 : 200, Abcam) at 4°C. Next, cells were incubated with the secondary antibody IgG (ab6721, 1 : 1000, Abcam) at 37°C for 30 min, followed by color-developing with diaminobenzidine (Sigma Co., St. Louis, MO, USA) and counterstaining with hematoxylin for 1 min. Afterwards, cells were observed and photographed using an optical microscope (Eclipse E200, Nikon Co., Tokyo, Japan). The protein levels were analyzed with the help of Image-pro Plus software (Media Cybernetics, San Diego, CA, USA).

### 2.4. Cell Transfection

Two strands of small interfering (si) RNA targeting *lncRNA BANCR* (si-BANCR#1 and si-BANCR#2), *miR-15a-5p* inhibitor (miR-inhibitor), pcDNA3.1 vector-packaged overexpressed (oe)-TRIM59, and their negative controls were supplied by GenePharma (Shanghai, China). After Ect-ESCs were cultured in 6-well plates (3 × 10^5^cells/well) for 24 h, the above plasmids were transiently transfected into Ect-ESCs applying Lipofectamine 2000 (Thermo Fisher Scientific, Waltham, MA, USA) according to manufacturer's requirements.

### 2.5. Cell Counting Kit-8 (CCK-8) Assay

Ect-ESCs were placed in 96-well plates (3 × 10^3^cells/well) 24 h after cell transfection. At certain time points of cell culture (0, 24, 48, and 72 h), Ect-ESCs were incubated with 10 *μ*L of CCK-8 solution at 37°C for 1 h. After that, the absorbance at a wavelength of 450 nm was measured on a microplate reader (Bio-Rad, Hercules, CA, USA).

### 2.6. Colony Formation Assay

Ect-ESCs were placed into 6-well plates (500 cells/well) for 10 days of cell culture. After that, Ect-ESCs were fixed with 4% paraformaldehyde and stained with 0.1% crystal violet solution (Sigma, St. Louis, MO, USA) for 20 min. Next, the plate was washed with PBS and dried, and the number of colonies in each well was counted and analyzed.

### 2.7. Transwell Assay

The invasion potential of Ect-ESCs was analyzed using transwell assay. Simply put, the apical transwell chamber was pre-coated with Matrigel (BD Biosciences, San Jose, CA, USA), followed by cell seeding (4 × 10^4^cells/0.1 mL). Then, the basolateral transwell chamber was supplemented with DMEM supplemented with 10% FBS. After 24 h of cell culture, cells were stained with crystal violet. Non-invasive cells were wiped out, and the number of invasive cells was counted under a microscope.

### 2.8. Real-Time-Quantitative Polymerase Chain Reaction (RT-qPCR)

After extraction of the total RNA content in cells and tissues using the TRIzol reagent (Thermo Fisher Scientific, Waltham, MA, USA), the extracted RNA was reverse-transcribed into the complementary DNA using RT-PCR assay kits (K1621, Thermo Fisher Scientific, Waltham, MA, USA). The quantification of gene expression was achieved by real-time PCR. The relative amount of gene expression was quantified according to the 2^-*ΔΔ*Ct^ method, with GAPDH and U6 as endogenous references [28]. The primers used in RT-qPCR are shown in Table 1.

### 2.9. Western Blot Assay

The total protein samples were extracted from tissues or cells using radioimmunoprecipitation assay buffer. According to the instructions of the producer, the protein concentration was determined using the bicinchoninic acid protein assay kit (ab102536, Abcam, Cambridge, UK). An equal amount of protein (30 *μ*g) was separated by 10% sodium dodecyl sulfate-polyacrylamide gel and transferred onto polyvinylidene fluoride membranes. After blockade with Tris-HCl buffered saline containing 0.1% Tween-20 and 5% skim milk, membranes were incubated with primary antibodies against TRIM59 (ab166793, 1 : 500, Abcam) and GAPDH (ab181602, 1 : 10000, Abcam) overnight and then with secondary antibody against IgG (ab6721, 1 : 2000, Abcam) for 1 h. Membranes were visualized using the enhanced chemiluminescence-PLUS (Amersham Biosciences, Sweden). Eventually, the grayscale was quantified using Image-Pro Plus (Media Cybernetics, San Diego, CA, USA).

### 2.10. Bioinformatics

The subcellular localization of *lncRNA BANCR* was predicted on the lncLocator database (http://www.csbio.sjtu.edu.cn/bioinf/lncLocator/?tdsourcetag=s_pcqq_aiomsg) [29]. The downstream miRNAs of *lncRNA BANCR* (Table S1) and the binding sites of *lncRNA BANCR* to *miR-15a-5p* were predicted on the RNA22 database (https://cm.jefferson.edu/rna22/Interactive/) [30]. The downstream target genes of *miR-15a-5p* were predicted on Starbase (http://starbase.sysu.edu.cn/) [31] and miRWalk (http://mirwalk.umm.uni-heidelberg.de/) [32] databases (Table S2-S3). The binding sites of *miR-15a-5p* to TRIM59b were predicted on the Starbase database.

### 2.11. Subcellular Fractionation Assay

Following the manufacturer's protocol, the nucleus and cytoplasm were separated using PARIS kits (AM1921, Thermo Fisher Scientific). In brief, Ect-ESCs were collected, lysed with the preparation buffer, and then centrifuged. The fractionation of the supernatant and nuclear pellet was achieved using the cell disruption buffer, and the nuclear pellet was preserved for RNA extraction and analysis. Lastly, *lncRNA BANCR* was analyzed by RT-qPCR, with U6 (the control of nucleus) and GAPDH (the control of cytoplasm) as internal references.

### 2.12. Dual-Luciferase Assay

The synthesized *BANCR* fragments containing the binding sites to *miR-15a-5p* and *BANCR* fragments containing the mutant binding sites to *miR-15a-5p*, as well as TRIM59 fragments containing the binding sites to *miR-15a-5p* and TRIM59 fragments containing the mutant binding sites to *miR-15a-5p* were inserted into pmirGLO-reporter plasmids. The above-constructed luciferase reporter plasmids (*BANCR*-WT or *BANCR*-MUT, TRIM59-WT or TRIM59-MUT) were co-transfected into Ect-ESCs with mimics-NC or *miR-15a-5p* mimics (miR-mimics). After 48 h, cells were harvested and lysed, and the luciferase activity was quantified using the luciferase assay kits (16186, Thermo Fisher Scientific). All plasmids were supplied by GenePharma.

### 2.13. Statistical Analysis

Data analysis and graphing were conducted with the help of SPSS21.0 software (IBM Corp, Armonk, NY, USA) and GraphPad Prism 8.0 software (GraphPad Software Inc., San Diego, CA, USA). Measurement data were formalized as mean ± standard deviation (SD). First, data were subjected to normality and homogeneity of variance tests, which revealed that data conformed to normal distribution and homogeneity of variance. Then, pairwise comparisons were analyzed using the *t* test, and multi-group comparisons were analyzed using one-way or two-way analysis of variance (ANOVA), followed by Tukey's multiple comparison test. The *P* values were attained by two-sided tests, and a value of *P* < 0.01 was indicative of extremely statistical significance.

## 3. Results

### 3.1. Silencing lncRNA BANCR Suppresses Ect-ESC Proliferation and Invasion

**Figure 1:**
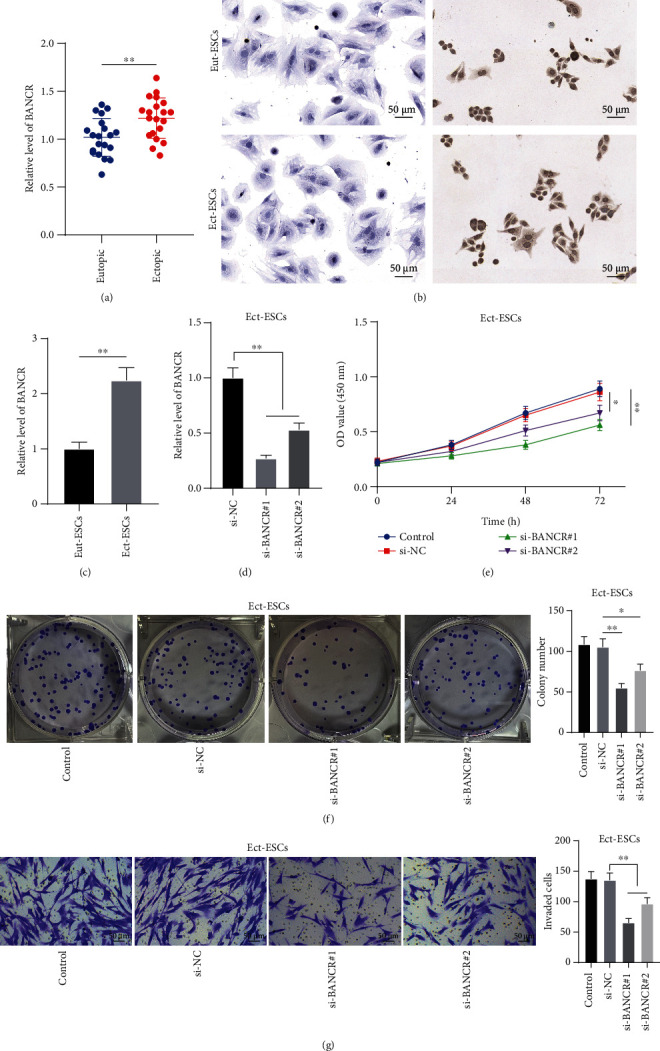
Silencing *lncRNA BANCR* suppresses ectopic endometrial stromal cell proliferation and invasion. (a) *LncRNA BANCR* expressions in eutopic and ectopic endometrial tissues (*N* = 20) were determined by RT-qPCR; (b) CK19 and vimentin expressions were determined by immunocytochemistry; (c) *LncRNA BANCR* expressions in eutopic and ectopic endometrial stromal cells (Eut-ESCs/Ect-ESCs) were determined by RT-qPCR. Ect-ESCs were transfected with si- BANCR (si-BANCR#1 and si-BANCR#2), with transfection of si-NC as the negative control. (d) Knockdown efficiency of si-BANCR was determined by RT-qPCR; (e, f) Proliferation potential of Ect-ESCs was assessed by CCK-8 (e) and colony formation (f) assays; (g) Invasion potential of Ect-ESCs was assessed by transwell assay. Cell experiments were performed thrice independently. Data in figures (c)-(d) were formalized as mean ± SD. Data in figures (a) and (c) were analyzed using the *t* test, data in figures (d), (f), and (g) were analyzed using one-way ANOVA, and data in figure (e) were analyzed using two-way ANOVA, followed by Tukey's multiple comparison test. ∗∗*P* < 0.01.

A previous study has documented the upregulation of *lncRNA BANCR* in EM [11]. First, we determined the expression levels of *lncRNA BANCR* in eutopic and ectopic endometrial tissues and found that *lncRNA BANCR* was upregulated in ectopic endometrial tissues (*P* < 0.05, Figure 1(a)). Then, we isolated ESCs from eutopic and ectopic endometrial tissues. Through immunocytochemistry, it was found that cytoskeleton protein CK19 was negatively expressed and Vimentin was positively expressed (Figure 1(b)), indicating the successful extraction of ESCs. Accordingly, the subsequent experiment revealed higher expression levels of *lncRNA BANCR* in Ect-ESCs relative to eutopic ESCs (*P* < 0.05, Figure 1(c)). Several studies have documented that suppression of abnormal proliferation or invasion of ESCs mitigates EM progression [33–35]. To further explore the role of *lncRNA BANCR* in ESC proliferation and invasion, Ect-ESCs were transfected with si-BANCR (si-BANCR#1 and si-BANCR#2) to downregulate *lncRNA BANCR* expression (*P* < 0.05, Figure 1(d)). It was found that the proliferation potential of Ect-ESCs was significantly reduced upon silencing *lncRNA BANCR* (*P* < 0.05, Figure 1(e)-1(f)), and so was the invasion potential of Ect-ESCs (*P* < 0.05, Figure 1(g)). Altogether, the preceding data suggested that silencing *lncRNA BANCR* suppressed Ect-ESC proliferation and invasion.

### 3.2. LncRNA BANCR Targets and Inhibits miR-15a-5p

**Figure 2:**
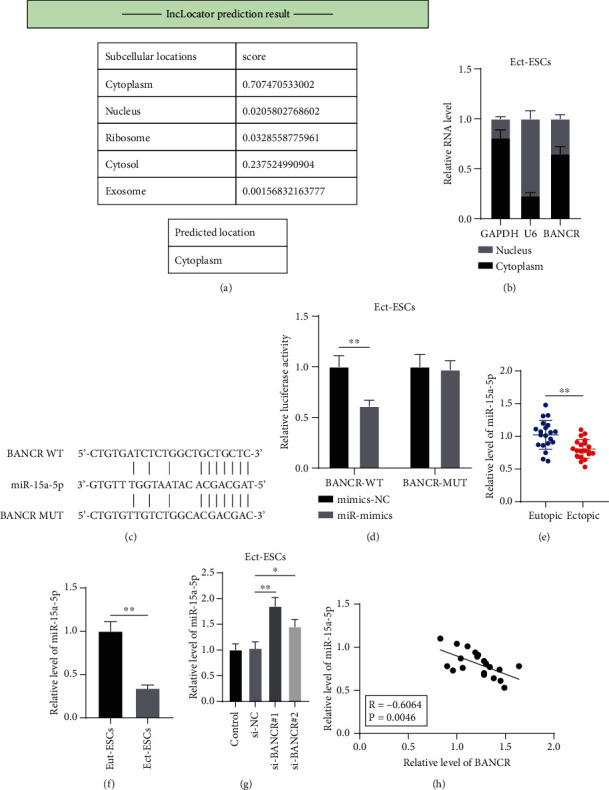
*LncRNA BANCR* targets and inhibits *miR-15a-5p*. (a) Subcellular localization of *lncRNA BANCR* was predicted on the lncLocator database; (b) Subcellular localization of *lncRNA BANCR* was testified by the subcellular fractionation assay; (c) Binding sites of *lncRNA BANCR* to *miR-15a-5p* were predicted on the RNA22 database; (d) Binding relationship between *lncRNA BANCR* and *miR-15a-5p* was verified by the dual-luciferase assay; (e) *miR-15a-5p* expressions in eutopic and ectopic endometrial tissues (*N* = 20) were determined by RT-qPCR; (f)—(g) *miR-15a-5p* expressions in Eut-ESCs and Ect-ESCs were determined by RT-qPCR; (h) Correlation between *lncRNA BANCR* and *miR-15a-5p* in ectopic endometrial tissues of EM patients (*N* = 20) was analyzed using Pearson correlation analysis. Cell experiments were performed thrice independently. Data in figures (b), (d), (f), and (g) were formalized as mean ± SD. Data in figure (d) were analyzed using two-way ANOVA, data in figures (e)–(f) were analyzed using the *t* test, and data in figure (g) were analyzed using one-way ANOVA, followed by Tukey's multiple comparison test. ∗∗ *P* < 0.01.

To explore the downstream mechanism of *lncRNA BANCR*, we predicted the subcellular localization of *lncRNA BANCR* on the lncLocator database and found that *lncRNA BANCR* was predominantly expressed in the cytoplasm (Figure 2(a)). The subcellular fractionation assay further validated that *lncRNA BANCR* was mainly expressed in the cytoplasm of Ect-ESCs (*P* < 0.05, Figure 2(b)). *LncRNA BANCR* as a ceRNA can act as a sponge of miRNA [14]. Next, the downstream miRNAs of *lncRNA BANCR* were predicted on the RNA22 database. Among these, *miR-15a-5p* is documented to be downregulated in EM [18]. According to the predicted binding sites of *lncRNA BANCR* to *miR-15a-5p* (Figure 2(c)), the dual-luciferase assay was performed to testify the binding relationship between *lncRNA BANCR* and *miR-15a-5p* (*P* < 0.05, Figure 2(d)). *miR-15a-5p* expression levels in ectopic endometrial tissue and Ect-ESCs were lower than those in eutopic endometrial tissue and eutopic endometrial stromal cells (*P* < 0.05, Figure 2(e)-2(f)). *miR-15a-5p* was significantly upregulated by silencing *lncRNA BANCR* (*P* < 0.05, Figure 2(g)). In addition, *lncRNA BANCR* was negatively correlated with *miR-15a-5p* in 20 cases of EM patients (*P* < 0.05, Figure 2(h)). Altogether, the preceding data suggested that *lncRNA BANCR* targeted and inhibited *miR-15a-5p*.

### 3.3. miR-15a-5p Downregulation Reverses the Effects of Silencing lncRNA BANCR on Inhibiting Ect-ESC Proliferation and Invasion

**Figure 3:**
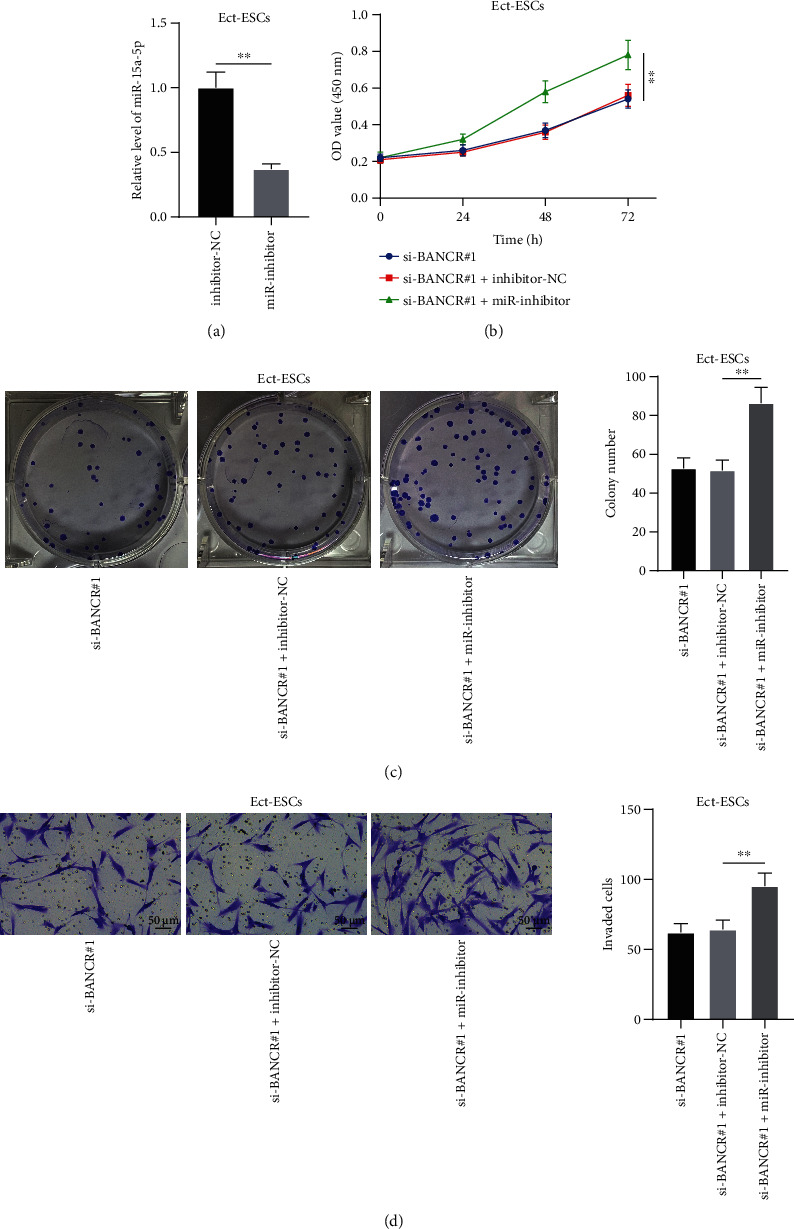
*miR-15a-5p* downregulation reverses the effects of silencing *lncRNA BANCR* on inhibiting ectopic endometrial stromal cell proliferation and invasion. Ect-ESCs were transfected with *miR-15a-5p* inhibitor (miR-inhibitor), with transfection of inhibitor-NC as the negative control. (a) Knockdown efficiency of miR-inhibitor was determined by RT-qPCR; (b)–(c) Proliferation potential of Ect-ESCs was assessed by CCK-8 (b) and colony formation (c) assays; (d) Invasion potential of Ect-ESCs was assessed by transwell assay. Cell experiments were performed thrice independently. Data in figures (a)–(d) were formalized as mean ± SD. Data in figure (a) were analyzed using the *t* test, data in figure (b) were analyzed using two-way ANOVA, and data in figures (c)–(d) were analyzed using one-way ANOVA, followed by Tukey's multiple comparison test. ∗∗ *P* < 0.01.

To probe the role of *miR-15a-5p* in Ect-ESC proliferation and invasion, Ect-ESCs were transfected with *miR-15a-5p* inhibitor (miR-inhibitor) to downregulate *miR-15a-5p* expression (*P* < 0.05, Figure 3(a)), followed by combined treatment with si-BANCR#1 which had better knockdown effectiveness. Through CCK-8 and colony formation assays, it was found that the proliferation potential of Ect-ESCs was enhanced upon *miR-15a-5p* downregulation (*P* < 0.05, Figure 3(b)-3(c)). Through transwell assay, it was observed that the invasion potential of Ect-ESCs was also upregulated upon *miR-15a-5p* downregulation (*P* < 0.05, Figure 3(d)). Altogether, the preceding data suggested that *miR-15a-5p* downregulation reversed the effects of silencing *lncRNA BANCR* on inhibiting Ect-ESC proliferation and invasion.

### 3.4. miR-15a-5p Targets and Inhibits TRIM59 Expression

**Figure 4:**
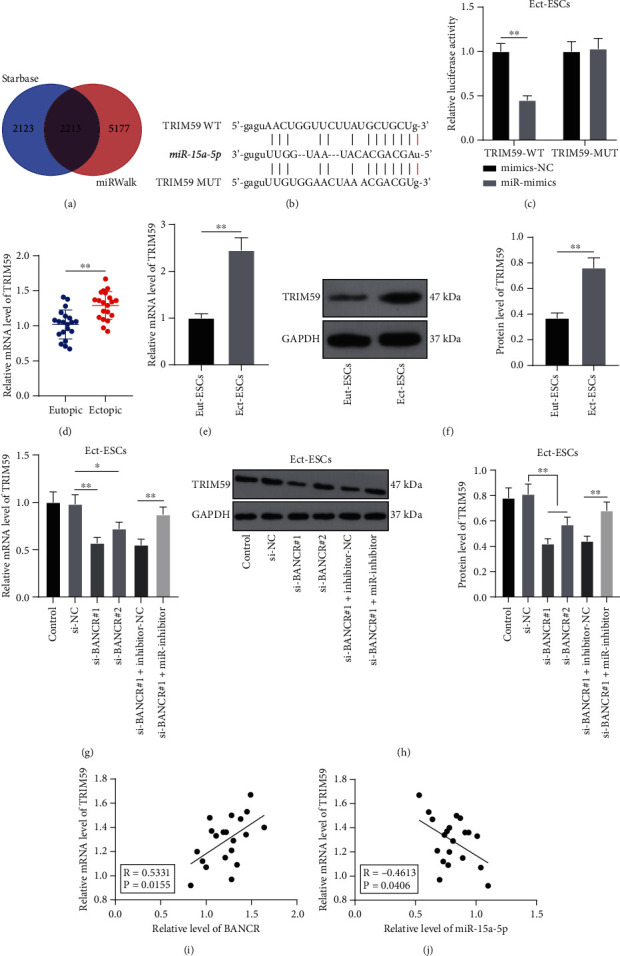
*miR-15a-5p* targets and inhibits TRIM59. (a) Downstream target genes of *miR-15a-5p* were predicted on the Starbase and miRWalk databases and intersections were identified; (b) Bindings sites of *miR-15a-5p* to TRIM59 were predicted on the Starbase database; (c) Binding relationship between *miR-15a-5p* and TRIM59 in Ect-ESCs was testified by the dual-luciferase assay; (d) Levels of TRIM59 in eutopic and ectopic endometrial tissues (*N* = 20) were determined by RT-qPCR; (e)–(f) Expression levels of TRIM59 in Eut-ESCs and Ect-ESCs were determined by RT-qPCR (e) and Western blot assay (f); (g)–(h) Expression levels of TRIM59 in Ect-ESCs were determined by RT-qPCR (g) and Western blot assay (h); (i) Correlation between *lncRNA BANCR* and TRIM59 in ectopic endometrial tissues of EM patients (*N* = 20) was analyzed by Pearson correlation analysis; (j) Correlation between *miR-15a-5p* and TRIM59 in ectopic endometrial tissues of EM patients (*N* = 20) was analyzed by Pearson correlation analysis. Cell experiments were performed thrice independently. Data in figures (c), (e), and (h) were formalized as mean ± SD. Data in figure (c) were analyzed using two-way ANOVA, data in figures (d)–(f) were analyzed using the *t* test, and data in figures (g)–(h) were analyzed using one-way ANOVA, followed by Tukey's multiple comparison test. ∗∗ *P* < 0.01.

To further explore the downstream mechanism of *miR-15a-5p*, the downstream target genes of *miR-15a-5p* were predicted on the Starbase and miRWalk databases, and our attention was paid to TRIM59 (Figure 4(a)). In a previous study, TRIM59 was elevated in EM [26]. Then, according to the binding sites of *miR-15a-5p* to TRIM59 predicted on the Starbase database (Figure 4(b)), the dual-luciferase assay was performed to testify the binding relationship between *miR-15a-5p* and TRIM59 (*P* < 0.05, Figure 4(c)). Next, we determined the expression levels of TRIM59 in tissues and cells and found that TRIM59 expression levels were augmented in ectopic endometrial tissues and Ect-ESCs (*P* < 0.05, Figure 4(d)-4(f)), declined by silencing *lncRNA BANCR*, and upregulated in response to *miR-15a-5p* downregulation (*P* < 0.05, Figure 4(g)-4(h)). Besides, TRIM59 mRNA level was positively correlated with *lncRNA BANCR* (*P* < 0.05, Figure 4(i)) and negatively correlated with *miR-15a-5p* (*P* < 0.05, Figure 4(j)) in 20 cases of EM patients. Altogether, the preceding data suggested that *miR-15a-5p* targeted and inhibited TRIM59 expression.

### 3.5. TRIM59 Overexpression Reverses the Effects of Silencing lncRNA BANCR on Inhibiting Ect-ESC Proliferation and Invasion

**Figure 5:**
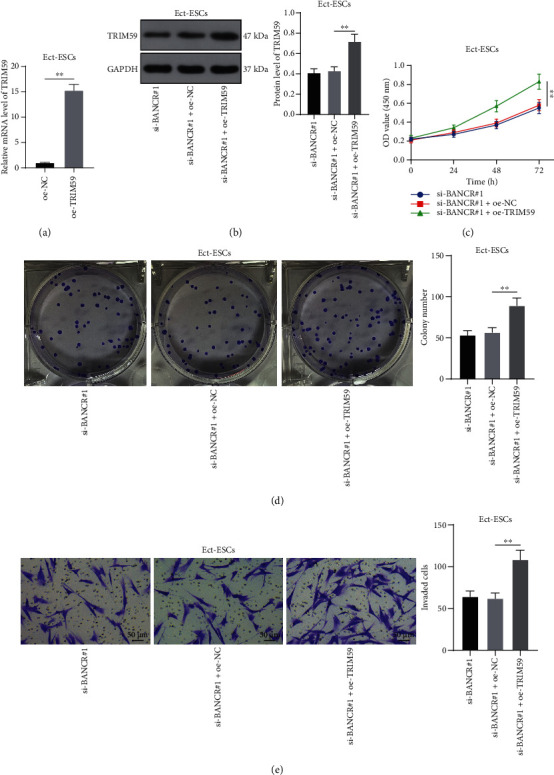
TRIM59 overexpression reverses the effects of silencing *lncRNA BANCR* on inhibiting ectopic endometrial stromal cell proliferation and invasion. Ect-ESCs were transfected with oe-TRIM59, with transfection of oe-NC as the negative control. (a)–(b) Expression levels of TRIM59 were determined by RT-qPCR (a) and Western blot assay (b); (c)–(d) Proliferation potential of Ect-ESCs was assessed by CCK-8 (c) and colony formation (d) assays; (e) Invasion potential of Ect-ESCs was assessed by transwell assay. Cell experiments were performed thrice independently. Data in figures (a)–(e) were formalized as mean ± SD. Data in figure (a) were analyzed using the *t* test, data in figures (b), (d), and (e) were analyzed using one-way ANOVA, and data in figure (c) were analyzed using two-way ANOVA, followed by Tukey's multiple comparison test. ∗∗ *P* < 0.01.

To evaluate the role of TRIM59 in Ect-ESC proliferation and invasion, Ect-ESCs were transfected with oe-TRIM59 to upregulate TRIM59 expression (*P* < 0.05, Figure 5(a)-5(b)), followed by combined treatment with si-BANCR#1 which had better knockdown effectiveness. Our subsequent experiments revealed that TRIM59 overexpression enhanced the proliferation potential of Ect-ESCs (*P* < 0.05, Figure 5(c)-5(d)), as well as the invasion potential of Ect-ESCs (*P* < 0.05, Figure 5(e)). Altogether, the preceding data suggested that TRIM59 overexpression reversed the effects of silencing *lncRNA BANCR* on inhibiting Ect-ESC proliferation and invasion.

## 4. Discussion

EM is a gynecological disorder with malignant potential and remains a challenge in the clinic due to the lack of therapies with curable efficacy [7]. ECSs are identified as the major resident cells in the endometrium and alterations in their behaviors, such as proliferation, invasion, migration, and epithelial-mesenchymal transition, are associated with the pathogenesis of EM [33, 36, 37]. In addition, lncRNAs are involved in gene regulation in EM and the alteration of lncRNA expression level affects EM progression [38]. Apart from lncRNAs, miRNAs and circRNAs also play crucial roles in the regulation of EM progression [39–41]. In the present study, our findings supported that *lncRNA BANCR* promotes Ect-ESC proliferation and invasion via the *miR-15a-5p*/TRIM59 axis.

*LncRNA BANCR* is a putative biomarker of cell malignancy [9, 10]. A pioneering study by Zhu and colleagues has uncovered that knockdown of *lncRNA BANCR* restrains angiogenesis in ectopic endometrial tissues by targeting the extracellular signal-regulated kinase/mitogen-activated protein kinase signaling pathway [11]. In this study, augmented *lncRNA BANCR* levels were evident in ectopic endometrial tissues of EM patients. Then, ESCs were isolated from eutopic and ectopic endometrial tissues and it was found that *lncRNA BANCR* was prominently expressed in Ect-ESCs. To further evaluate the functionality of *lncRNA BANCR* in Ect-ESC proliferation and invasion, *lncRNA BANCR* was silenced in Ect-ESCs using si-BANCR, upon which the proliferation and invasion potentials of Ect-ESCs were declined. It might be correlated with the effects of *lncRNA BANCR* on promoting the expression levels of ESCs mobility-related proteins, such as matrix metalloproteinase 1 and 2 [42–44]. Therefore, it is plausible to suggest that silencing *lncRNA BANCR* suppresses Ect-ESC proliferation and invasion.

LncRNAs further function as the sponges of miRNAs to play performance in human diseases [45, 46]. miRNAs can serve as desirable biomarkers for EM diagnosis and treatment due to their stability in body fluids, specific and distinct signatures relative to clinical controls, and biological relevance to EM pathogenesis [47]. To further analyze the downstream mechanism of *lncRNA BANCR*, the lncLocator database and subcellular fractionation assay validated the cytoplasmic localization of *lncRNA BANCR* in Ect-ESCs, suggesting the possibility of *lncRNA BANCR* as a ceRNA in Ect-ESCs. A prior microarray analysis disclosed the downregulation of *miR-15a-5p* in endometriotic tissues and its association with angiogenesis-related proteins [48]. In addition, *miR-15a-5p* was previously demonstrated to repress proliferation, migration, and invasion of Ect-ESCs and mobility and angiogenesis of endometrial mesenchymal stem cells [18, 20], further highlighting the participation of *miR-15a-5p* in EM pathogenesis. Accordingly, the dual-luciferase assay verified the binding relationship between *lncRNA BANCR* and *miR-15a-5p*, and decreased *miR-15a-5p* expression levels were found in ectopic endometrial tissues and Ect-ESCs and elevated in response to silencing *lncRNA BANCR*, suggesting that *lncRNA BANCR* negatively regulated *miR-15a-5p* in EM. Subsequently, *miR-15a-5p* was downregulated in Ect-ESCs in the si-BANCR group using the *miR-15a-5p* inhibitor, upon which the proliferation and invasion potentials of Ect-ESCs were enhanced. In accordance, *miR-15a-5p* was capable of improving pro-apoptosis B-cell lymphoma 2-associated X protein (Bcl2) and decreasing anti-apoptosis B-cell lymphoma 2 (Bax) wherein the ratio of Bax/Bcl2 is associated with apoptosis of ESCs [49, 50]. Collectively, the above findings and shreds of evidence initially demonstrated that *lncRNA BANCR* promotes Ect-ESC proliferation and invasion by inhibiting *miR-15a-5p*.

Furthermore, the subsequent bioinformatic data directed our attention to TRIM59. As indicated by a previous study, TRIM59 functioned as a positive regulator of Ect-ESC invasion [26]. The binding relationship between *miR-15a-5p* and TRIM59 was testified by the dual-luciferase assay. Meanwhile, increased TRIM59 expression levels were found in ectopic endometrial tissues and Ect-ESCs, downregulated by silencing *lncRNA BANCR* and upregulated by inhibition of *miR-15a-5p*, indicative of a positive correlation between *lncRNA BANCR* and TRIM59 and a negative correlation between *miR-15a-5p* and TRIM59. To confirm the effects of TRIM59 on Ect-ESC proliferation and invasion, TRIM59 was overexpressed in Ect-ESCs in the si-BANCR group using oe-TRIM59, upon which Ect-ESC proliferation and invasion were accelerated. Likewise, TRIM59 acts as a booster of proliferation and invasion of endometriosis-associated cancer, such as ovarian, breast, and cervical cancers [51–53]. Altogether, the above findings initially supported that *lncRNA BANCR* promotes Ect-ESC proliferation and invasion by inducing TRIM59 upregulation.

## 5. Conclusion

To conclude, our study was the first of its kind that unveiled the promotive role of the *lncRNA BANCR*-mediated ceRNA network in Ect-ESC proliferation and invasion and provided a novel theoretical reference for the clinical study of *lncRNA BANCR* in EM. However, whether other downstream miRNAs of *lncRNA BANCR* and downstream target genes of *miR-15a-5p* are involved in functions of Ect-ESCs remains unknown. In the next step, experimentation will be designed to investigate other downstream mechanisms of *lncRNA BANCR* and *miR-15a-5p* in Ect-ESCs and the upstream mechanism of *lncRNA BANCR* in Ect-ESCs.

## Figures and Tables

**Table 1:** qPCR primers.

Gene	Forward primer (5'‐3')	Reverse primer (5'‐3')
BANCR	ACCTGAATCTCACCTCTGCAA	GCCAGGGATGACTTGCGTAT
miR-15a-5p	TCGGCAGGTAGCAGCACATA	CTCAACTGGTGTCGTGGA
TRIM59	CGTGTACTGCCATGCTCTCA	CCAACATCACAGAGAGCCGT
GAPDH	GATGCTGGCGCTGAGTACG	GCTAAGCAGTTGGTGGTGC
U6	CTCGCTTCGGCAGCACATA	AACGATTCACGAATTTGCGT

## Data Availability

The data that support this study are available from the corresponding author upon reasonable request.

## Supplementary Materials

Supplementary Materials Table S1: The downstream miRNAs of *lncRNA BANCR* were predicted on the RNA22 database. Table S2: The target genes of *miR-15a-5p* were predicted on the StarBase database. Table S3: The target genes of *miR-15a-5p* were predicted on the miRWalk database.
